# Multiple needs and multiple treatments. What's a clinician to do? Update on the psychosocial treatment of disruptive behaviours in childhood

**DOI:** 10.1097/YCO.0000000000000823

**Published:** 2022-09-19

**Authors:** Brendan F. Andrade, Madison Aitken, Sabrina Brodkin, Vilas Sawrikar

**Affiliations:** a Margaret and Wallace McCain Centre for Child Youth and Family Mental Health, Centre for Addiction and Mental Health; b Department of Psychiatry, University of Toronto, Toronto, Canada; c School of Health in Social Science, University of Edinburgh, UK; d Cundill Centre for Child and Youth Depression, Centre for Addiction and Mental Health; e Ontario Institute for Studies in Education, University of Toronto, Toronto Canada

**Keywords:** disruptive behaviour, psychosocial treatment, treatment selection

## Abstract

There are a wide range of psychosocial treatment options, delivered in different modalities, for children with disruptive behaviour. However, clinicians face many challenges in ensuring the empirically supported treatments (ESTs) they select will be effective for their patient. This has prompted studies to generate knowledge on how to improve treatment outcomes for children with disruptive behaviour. This review identifies the major challenges in treatment selection as well as emerging research seeking to improve outcomes.

**Recent findings:**

This review emphasizes the salience of the research-practice gap associated with establishing ESTs using narrow definitions of clinical problems. Recent research is reviewed considering the complex determinants of disruptive behaviours, including parent and family factors that influence outcomes. The review subsequently outlines recent advances in research and clinical practice guidelines aiming to surmount these challenges. Key advances discussed include examining the most impactful components of ESTs, personalizing interventions by targeting core dysfunction underlying behaviour, and addressing parent factors including mental health and cultural relevance to improve outcomes.

**Summary:**

Thorough assessment of patients’ needs, combined with knowledge of treatment response predictors, are recommended to determine the most suitable treatment plan. Recent advances have focused on developing and designing interventions that meet needs in a way that is flexible and tailored.

## INTRODUCTION

Children with disruptive behaviour represent about 6% of the general population and experience tremendous, short-term and long-term social, academic and family morbidity [1,2]. Although impairments because of disruptive behaviour exist in the absence of a diagnostic label, many of these children are diagnosed with Oppositional Defiant Disorder (ODD) or Conduct Disorder and frequently have co-occurring neurodevelopmental disorders, such as Attention-Deficit Hyperactivity Disorder (ADHD) and Learning Disorders [3]. Moreover, the presence of developmentally inappropriate disruptive behaviour in childhood increases the odds of adolescent and adult severe mental illness compared with nonaffected peers [4^▪^,5^▪^,^6^,^7^]. This review presents recent research findings relevant to improving the effectiveness of psychosocial intervention to prevent these tremendous impairments. 

**Box 1:**
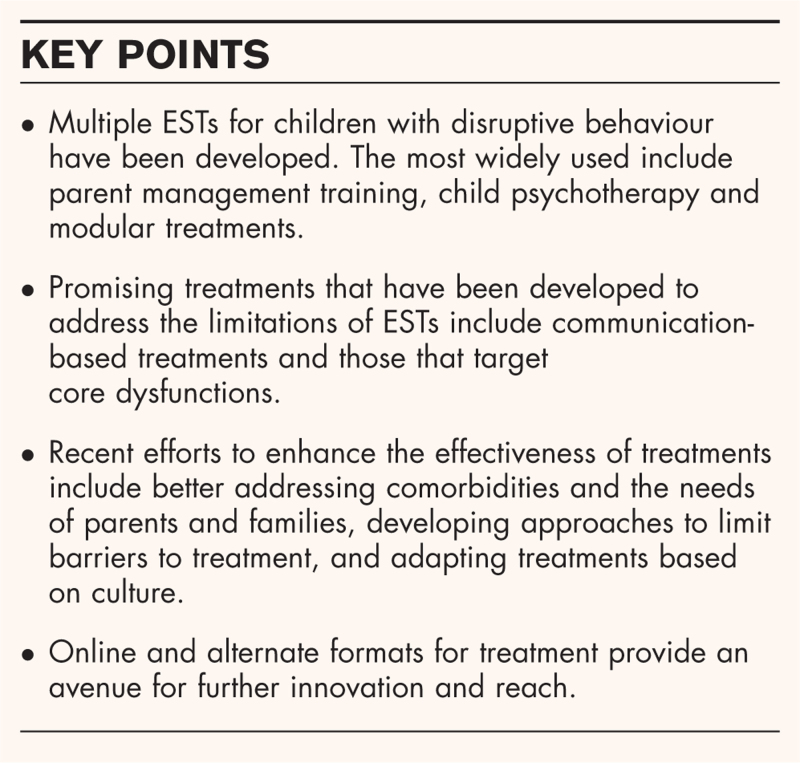
no caption available

## CURRENT RECOMMENDED TREATMENTS FOR CHILDREN WITH DISRUPTIVE BEHAVIOUR

A recent systematic review of clinical practice guidelines for the assessment, prevention and treatment of disruptive behaviour identified the National Institute for Health and Care Excellence (NICE) guideline for ‘Antisocial behaviour and conduct disorders in children and young people’ as having a high-quality rating, meaning it was rigorously developed and has the potential to facilitate clinically significant improvements [8,9]. Accordingly, we recommend that clinicians refer to this guideline when developing a plan for assessment and treatment of children with disruptive behaviour. Empirically supported psychosocial treatments (ESTs) identified in the NICE guideline include group and individual parent-training programs, parent and child-training programs, and child-focused programs. [10].

One of the most established ESTs for childhood disruptive behaviour is Parent Management Training (PMT), also known as Behavioural Parent Training (BPT), parent skills training, and other similar labels [11,12]. It is considered a front-line treatment for children from early-to-middle childhood [13]. PMT involves psychoeducation and parent coaching to further develop effective parenting skills based on behavioural principles [14]. There are a number of well evaluated PMTs to choose from based on specific need. Some are delivered in groups such as Triple P – Positive Parenting Program [15,16] and Incredible Years [17], whereas others are held individually like Helping the Noncompliant Child (HNC [18]). Other popular PMTs include Parent Management Training Oregon Model (PMTO [19]) and Parent–Child Interaction Therapy (PCIT [20,21]).

ESTs that focus on the child are grounded in cognitive behavioural therapy (CBT) and help children better understand and challenge social–cognitive biases, develop problem-solving skills and strengthen emotional and behavioural regulation skills. These treatments are most effective when combined with parenting treatment [22,23]. Additionally, these treatments have shown most promise with older children, and the NICE guidelines recommend starting child treatments at age 9 years. Some of the most widely used programs include Problem-Solving Skills Training (PSST [24]), Coping Power (CP [25,26]), Incredible Years-Dina Dinosaur [17], and Stop Now and Plan (SNAP [27]).

In addition to widely used ESTs, there are a number of promising intervention approaches to treat childhood disruptive behaviour. These treatments have undergone less rigorous research to support their efficacy or effectiveness; however, they are considered promising, given the studies that have been completed (see [1] for a more detailed review). Some of these programs emphasize an emotionally supportive approach to positive parenting to help children develop emotional and behavioural regulation skills. Examples of emotion focused, or emotion-enhanced intervention include Tuning In To Kids [28], Emotion Enhanced Triple-P [29] and PCIT Emotion Development [30]. Other promising programs involve strengthening parent–child communication. For example, the Collaborative and Proactive Solution (CPS) intervention helps parents identify situations that leads to challenging behaviour, determine if it is the result of a lagging skill in the child, and help parents and children work to actively solve the problem. Finally, the common element approach represents a promising treatment that may be especially useful for addressing comorbidity in children with disruptive behaviour. It is based on the concept that ESTs can be broken into components that target a specific need. A treatment plan can be created that includes components from different ESTs and selected based on the unique needs of a specific child. One example is the Modular Approach to Therapy for Children with Anxiety, Depression, Trauma or Conduct Problems (MATCH-ADTC [31,32^▪^]).

## MAJOR CHALLENGES

Clinicans who work with children with disruptive behaviour are faced with a number of clinical challenges. Indeed, research evidence indicates that ESTs for disruptive behaviour are not sufficiently effective for up to 50% of children, highlighting the burden on clinicians to deliver treatments that are not only empirically supported but also suitable for their patient's needs [33]. To that end, a major criticism of the research conducted to determine the status of ESTs and promising new treatments is that they reflect outcomes under controlled research conditions that may not reflect real-world clinical populations and settings, emphasizing the gap between research and practice [34]. This has prompted studies to generate knowledge to bridge the research–practice gap.

In line with this, recent research findings highlight that ESTs are typically tied to diagnostic subgroups of children that may not sufficiently capture the challenge of equifinality, or the idea that multiple causal pathways, or combinations of factors, can give rise to what is observed as disruptive behaviour [35]. Much research documents a range of factors related to disruptive behaviour, including deficits in emotion and behavioural self-regulation skills [36,37], mental abilities such as attention, impulsivity, working memory, planning, emotion regulation/reactivity processes [38–41], difficulties with social behaviour, biases in social information processing, and callous–unemotional traits [42,43]. As a practical example, while children A and B show disruptive behaviour, child A may have primary difficulties with regulating their emotion and reacting to mild provocation whereas child B misperceives others’ social intentions, reacts with aggression, and shows elevated callous–unemotional traits. In this simplified example, the key factors that underlie disruptive behaviour and possible targets for treatment differ for these children.

Defining clinical presentations for ESTs based on diagnostic criteria also reduces their applicability for children with comorbid mental health problems. The signficance of this challenge becomes apparent in real-world clinical settings where comorbidity is the rule rather than exception, with over half of children with one diagnosable disorder also having a second disorder [44,45]. However, the impact of comorbidity on treatment effectiveness is mixed and there is a growing body of research to show that commonly used treatments for disruptive behaviour are beneficial for children with comorbid conditions [46,47].

A final challenge of note is the appropriate consideration of parent and family factors when planning treatment for children with disruptive behaviour. Parents are important agents of change with regards to their children's treatment progress and most ESTs include a strong parent component. As a result, it is important that treatments for child disruptive behaviour consider parent factors that may influence engagement and treatment outcome. For example, parents of children with disruptive behaviour show high rates of mental illness themselves and experience disproportionate stress and adversity [48], which may influence parent engagement in treatment [49–51]. Parents may also experience practical barriers to participating in ESTs, such as childcare needs or availability in their geographic region [52,53]. In addition, parents’ perception of fit with their culture and beliefs may influence their engagement in ESTs for disruptive behaviour [54].

## RECENT ADVANCES IN TREATMENTS FOR DISRUPTIVE BEHAVIOUR IN CHILDHOOD

To improve outcomes, researchers have partially turned their attention to answering the longstanding question of ‘What treatment, for whom, is most effective, and under which set of circumstances?’ In line with this, below we describe the key themes emerging from contemporary research to optimize EST outcomes by addressing current challenges in the treatment of disruptive behaviour.

## IDENTIFYING KEY COMPONENTS OF INTERVENTION

Recent studies have begun to delineate the most impactful components of established treatment programs for disruptive behaviour [6,55,56^▪▪^]). For instance, two meta-analyses that systematically reviewed the components of parenting interventions indicated that techniques such as positive reinforcement, praise and natural and logical consequences were associated with stronger program effects, and behaviour management components had the highest likelihood of being effective in treatment settings [12,56^▪▪^]. Further, a recent microtrial showed that both antecedent-based (e.g. setting clear rules) and consequence-based strategies (e.g. praise, ignoring unwanted behaviour) decrease disruptive behaviour in children [57^▪^].

## TARGETING CORE DYSFUNCTIONS

Treatments are being developed to target the common and unique biobehavioural underpinnings of disruptive behaviour. These include transdiagnostic treatments that target underlying processes shared across diagnoses [45,46], as well as stratified interventions targeting underlying pathological and maintaining processes specific to subgroups with differential pathways to disruptive behaviour [34]. This is especially noteworthy for children with disruptive behaviour who often present with comorbid disorders and challenges, where targeting shared rather than specific causal processes may be critical to clinical success [58–61].

Research investigating the core dysfunction approach generally focuses on the putative utility of adapting ESTs according to individual differences in affective dimensions of problem behaviours. For example, new treatments are being developed to target irritability, anger, and deficits in emotion regulation underlying disruptive behaviour problems and comorbid emotional problems [23,62,63]. Recently developed treatments include the Unified Protocol for Transdiagnostic Treatment, CBT and integrated therapy with PMT [64–68]. Innovations in CBT targeting severe irritability, anger and aggression include behavioural exposure to frustrative nonrewarding and threatening stimuli [69]. Other treatments have expanded upon applications of emotion-coaching to improve child emotion regulation. For example, a recent school-based trial of Tuning Your Temper showed significant improvements in disruptive behaviour compared with a waitlist control condition [4^▪^,^70^]. Other recently developed interventions specifically target social–emotional deficits linked with callous–unemotional traits. For example, a randomized controlled trial showed that a modified PCIT program (PCIT Emotion Development) reduced callous unemotional traits along with oppositional defiant disorder and major depressive disorder symptoms [71]. Overall, the core dysfunction approach offers a useful way of personalizing intervention based on the interacting neurobiological and environmental factors contributing to children's disruptive behaviour.

## ADDRESSING PARENT FACTORS AND REDUCING BARRIERS TO TREATMENT TO IMPROVE OUTCOMES

The integral role of parents in the treatment of childhood disruptive behaviour has led to increased research focus on ways to address parent factors that may limit treatment engagement and benefit. Among the key parent-related barriers, studies have focused on parents’ own mental health, parental beliefs about factors contributing to the child's disruptive behaviour and treatment, ease of accessing ESTs and cultural adaptations.

### Addressing parent mental health

Some recent trials have targeted parent mental health directly. For example, a recent trial showed that an integrated intervention including BPT and CBT targeting mothers’ own depression symptoms [72^▪^] led to greater increases in positive parenting behaviour than standard BPT alone, mediated through increases in adaptive attributions for children's behaviour [72^▪^]. Other studies have focused on the effects of ESTs for disruptive behaviour on parents’ own mental health. For example, PCIT results in significant improvements in parent stress and depressive symptoms [73]. In addition, a recent meta-analysis of BPT showed that programs that included more content related to modifying antecedents of problem behaviour were associated with greater improvement in parents’ own mental health [74^▪▪^]. These recent trials suggest the possibility of addressing parent mental health as part of treatment of child disruptive behaviour.

### Parental beliefs about child problems and treatment

Researchers and clinicians have long posited that parents’ interpretations of the cause of their child's behaviour, or parental attributions, may affect how willing parents are to accept and engage in treatment [75]. For instance, if parents attribute the problem behaviour to the child, they may view a parent-directed treatment as less relevant compared with one that is child-directed. Likewise, parents who report low parental self-efficacy may feel less confident to implement the recommended positive parenting strategies prescribed by treatment. Thus, researchers have recently turned their attention to understanding the specific processes by which addressing parental attributions can lead to better child outcomes [76,77]. Findings suggest that parent-causal attributions may be particularly important in determining parent readiness for treatment, but that consideration of self-perceived positive parenting skills is key to understanding whether parent-causal attributions may be problematic for treatment readiness [78]. Once parents participate in treatment, child-responsible attributions that are resistant to change may negatively impact treatment outcomes through persistently negative parent–child communication [79]. These findings highlight the need for clinicians to assess, monitor and potentially address problematic parental attributions from the start and throughout treatment to ensure their potentially disruptive influence on treatment improvement is mitigated [80].

### Decreasing barriers to treatment access

Despite the increasing need for treatment of childhood-disruptive behaviour, access to effective mental health treatment remains persistently low. This has been attributed in part to a reliance on in-person models of treatment delivery that require significant parent time commitment. Two alternate intervention formats have been investigated with the aim of increasing access: online interventions; and brief interventions.

The coronavirus disease 2019 (COVID-19) pandemic, and requirements for social distancing, have accelerated online treatment delivery models. The majority of these programs focus on PMT and are made up of vignettes, role-plays, and didactic elements [5^▪^,^81^–^86^]. Some include a live component using video conferencing technology that can include parent–child coaching [81,85,87]. Research on the efficacy of these online options has largely found them to have similar results to in-person treatment, with some higher rates of drop out [5^▪^,^81^,^82^,^87^,^88^]. A recent trial of Triple P found that an online version of program, without clinician coaching, was not inferior to an in-person, clinician-delivered version [5^▪^]; however, this trial was not conducted in a clinic-referred sample, and further research is needed to test the noninferiority of online-delivered interventions in clinical samples, which generally have more complex or severe needs.

In addition to comparisons with in-person treatment, it is important to consider for whom online interventions may be most appropriate. There is some evidence that the parents who have historically been least likely to engage in PMT, such as fathers and single parents, are more likely to participate in online interventions [81]. In contrast, parents who experience more adjustment difficulties themselves benefit less from online programs [81,82].

The use of brief intervention formats has been suggested as another way to decrease wait times and improve engagement in ESTs [89]. Although some studies have reported significant reductions in child disruptive behaviour following only two or three PMT sessions [57^▪^,^90^] others have found that brief interventions result in smaller effects on disruptive behaviour compared with standard-length ESTs [82]. As a result, the potential for smaller effects must be weighed along with the potential benefits of increased engagement when considering brief intervention approaches.

### Increasing cultural relevance

There is increasing awareness of the importance of considering diversity in all of its forms to design and implement interventions that are equitable. Challenges with systemic bias, gender-bias and racism have particularly limited the relevance and reach of effective intervention for persons identifying as black, indigenous or persons of colour [91,92]. Efforts to increase the cultural relevance of ESTs for disruptive behaviour in diverse populations have focused on parent interventions. At the broader intervention development level, recent efforts have focused on comprehensive modifications, guided by stakeholder consultation to improve outcomes [93]. For example, Triple P follows a process of partnering with community stakeholders to identify adaptations that meet the community's needs [16,94]. Adaptations include delivery in community centres, providing culturally relevant rationales for strategies, and the omission of topics deemed less culturally relevant (e.g. token economy [95]). In general, available research has found that culturally adapted and standard ESTs have similar effects [96] and many comprehensively adapted interventions are at the acceptability-testing stage [95,97].

At the individual patient level, culturally informed assessment and personalized formulation approaches have been used to increase the cultural responsiveness of interventions. These approaches involve discussions with caregivers about their understanding of the reasons for their child's difficulties, and inquiries about cultural and identity factors that must be incorporated to ensure the treatment meets the family's needs [98,99^▪^]. The use of these individual, formulation-based approaches has shown promise in improving engagement of culturally diverse families in PMT. A benefit of these individual-level approaches is their flexibility and ease of deployment [99^▪^]; however, their flexibility also makes it challenging to operationalize and systematically test the effectiveness of intervention components. Given that many culturally adapted parenting programs have not undergone rigorous evaluation [100^▪▪^], additional research to determine their efficacy is needed.

## CONCLUSION

There are a wide range of treatment options, delivered in different modalities, for children with disruptive behaviour, many of which are empirically supported. The majority of these interventions are built with behavioural underpinning and target parents, children or systemic risk factors [22,23]. Although ESTs have been effective, there are challenges when children present with comorbid diagnoses or a complex set of symptoms, and when parents experience barriers to engaging in ESTs. These challenges have led to the development of some alternative treatment models that can address the diversity of needs [101]. As per NICE guidelines, clinicians should conduct a thorough assessment of their patients’ needs and combine that information with knowledge of treatment response predictors to determine the best treatment plan [10,102]. Recent advances have focused on developing and designing intervention that meets needs in a way that is flexible and tailored.

## Acknowledgements

*None.*

### Financial support and sponsorship

*None.*

### Conflicts of interest

*There are no conflicts of interest.*

## REFERENCES AND RECOMMENDED READING

Papers of particular interest, published within the annual period of review, have been highlighted as:

▪ of special interest
▪▪ of outstanding interest
